# Development of a bench system with capacitive sensor, sample compression, and TinyML for iron ore moisture measurement

**DOI:** 10.1038/s41598-025-26782-8

**Published:** 2025-11-28

**Authors:** Érica S. Pinto, Saulo N. Matos, Matheus Neiva, Gabriel A. Santos, Leandro S. Marcolino, Jó Ueyama, Thiago A. M. Euzébio, Gustavo Pessin, Philip V. Pritzelwitz, Alan Kardek Rêgo Segundo

**Affiliations:** 1 Programa de Pós-Graduação em Ciência da Computação, Universidade Federal de Ouro Preto, Ouro Preto, Minas Gerais Brasil; 2 Instituto de Ciências Matemáticas e de Computação, Universidade de São Paulo (ICMC-USP), São Carlos, Brasil; 3 School of Computing and Communications, Lancaster, UK; 4 Instituto Tecnológico Vale, Ouro Preto, Minas Gerais Brasil; 5 Programa de Pós-Graduação em Instrumentação, Controle e Automação de Processos de Mineração, Universidade Federal de Ouro Preto e Instituto Tecnológico Vale, Ouro Preto, Minas Gerais Brasil; 6 Helmholtz-Zentrum Dresden-Rossendorf, Dresden, Germany; 7 Virtus-CC, Campina Grande, Brasil; 8 College of Engineering and Computer Science, VinUniversity, Gia Lâm, Hanói, Vietnam

**Keywords:** Engineering, Electrical and electronic engineering

## Abstract

In the mineral sector, many processes use water for ore beneficiation processes. A lack of sensing or control of water content can lead to operational problems in various mineral processing operations, especially in ore transport. Current instrumentation systems are either slow or inaccurate. Therefore, a novel bench system was developed to address this gap by achieving a fast response time and improved accuracy. The developed instrument measures the ore moisture by using the real-dual-frequency method (RDFM) to assess the ore’s electrical conductivity and relative permittivity. Additionally, it takes into account the bulk density, the bench chamber level, and the compress torque. All these variables are used to create a tiny machine-learning (TinyML) model that evaluates the ore’s moisture with a low time response. This process is done while the ore sample is compressed to reduce air bubbles inside the samples and improve measurement. Experiments were performed using the bench system in a mining company’s physical analysis laboratory. The instrument was utilized to measure the moisture content in the ore, leading to the development of a dataset used to train and validate various tree-based tinyML models. The results indicate that ore compression enhances accuracy and that decision trees are effective for estimating moisture with a quicker response time.

## Introduction

An accurate measurement of process variables is essential across various industrial sectors. In ore mining operations, for example, the ore characteristics significantly impact stages such as exploration, processing, and transportation^1^. Among these characteristics, ore moisture is particularly important. Therefore, measuring the moisture content in iron ore is crucial for ensuring product quality and safety, determining pricing, and optimizing both processing and transportation operations^2^.

High-moisture ore materials can jeopardize the ore handling processes. Transporting, storing, and preparing the ore for processing becomes problematic when the ore is wet. For example, it can stick on the conveyor belt when transporting and even cause avalanches in feeders and piles^3^. Ore beneficiation processes are also affected. Moisture agglomerates fine ore particles, reducing the classification and comminution process efficiency^4^. Furthermore, it directly affects the transport of the final product by shipping. Millions of tons of iron ore are transported by sea annually, and the amount of water compromises the stability of ships to the point that there is a risk of capsize because of cargo liquefaction^2,5,6^.

Between 2006 and 2016, a minimum of eight reported incidents involving bulk carriers may have been caused by shifting iron ore cargo^6^. Depending on the ore type, values around 8.5 to 9.5% of water already represent a borderline percentage, as highlighted by Clout and Manuel^7^. This threshold can render sea transportation hazardous, compounding the issue of transporting excess water with no commercial value.

The inability to effectively manage the excess water can also negatively affect the commercial value of iron ore. Buyers and sellers of iron ore consider moisture content as a key factor in determining prices; generally, higher moisture content results in lower prices^2^.

The standard and most common method for determining moisture content in mining environments is the gravimetric method, also known as the drying oven method. This direct and absolute technique involves drying the material under test (MUT) in a temperature-controlled chamber at 105°C until it almost reaches a constant mass, which typically takes at least 4 hours^8,9^. Along with prolonged response times, this method is sample-destructive, highlighting the need for a more effective moisture measurement technique. In addition, this technique is labor-intensive, requiring personnel to weigh the samples before drying, place them in the oven, weigh them again after drying, and record the masses.

The current utilization of manual gravimetric drying methods for moisture determination in most mineral processing plants falls short in providing the necessary, timely, and accurate information essential for automatic control^10^. Researchers have suggested online methods to evaluate the material moisture. For instance, the use of microwave-based analyzers for moisture measurement^11,12^. However, using microwaves has limitations due to high costs, imprecision in industrial environments, and challenges in supporting, calibrating, and maintaining this type of equipment^13^.

There are also image-based approaches^14^. However, it lacks industrial validation. Cameras face challenges in industrial settings due to dust, rain, and poor lighting conditions, which negatively impact image processing accuracy^1,15^. Moreover, online methods can struggle to achieve high accuracy in practice due to variable presentation and sensor drift or degradation over time; consequently, plants often rely on more accurate offline systems.

Capacitive sensors are a viable option for measuring the moisture content of bulk materials by assessing the complex impedance of the MUT^16,17^. However, this technique is limited by its tendency to produce inaccurate measurements when the material contains air gaps. To overcome this issue, we propose an innovative bench system that efficiently measures the moisture content of iron ore samples with the bulk density standardized by the machine, allowing for moisture measurement in a compressed state. The system utilizes the real-dual-frequency method (RDFM) available in^18^ and Tiny Machine Learning (TinyML) to measure the complex impedance and the moisture of an ore sample. This approach aims to minimize the impact of air gaps on capacitive sensor readings. Specifically, RDFM is a validated and patented technique and technology^18,19^. It is used in soil moisture measurement systems to accurately determine relative permittivity and conductivity. Key process variables–conductivity, relative permittivity, density, torque, and chamber level–are used as inputs to the machine learning model to assess the ore’s moisture content of a sample accurately.

In this work, the developed instrumentation system was evaluated with iron ore samples containing moisture levels in the range of 7–9.5%, which corresponds to the critical interval for safe handling and transport. For the ore samples used in this research, moisture levels above 8.5% pose a liquefaction risk during shipping operations, highlighting the importance of accurate measurement in this range, especially since the critical threshold depends on the mineral type.

There is a lack of measurement equipment available to meet the industry demand for rapid, on-site determination of ore moisture with precision and accuracy comparable to the standard method, yet with short response times. This gap affects multiple stages of processing and transport; however, in this study, we focus on the shipping stage, where pre-shipment testing is critical due to cargo-liquefaction risk. In this context, adherence to the Transportable Moisture Limit (TML) is mandatory, underscoring the need for timely and reliable measurement.

To this end, we conducted experiments using the developed method and benchtop instrument at the Physical Analysis Laboratory located at Vale SA, Ponta da Madeira, São Luís, Maranhão, Brazil. As one of the leading mining companies in Brazil, Vale SA operates the Ponta da Madeira Maritime Terminal (TMPM), a port primarily designed for exporting iron ore, manganese, and pellets from its mines. These materials are then loaded into ships for transportation to various international destinations.

The equipment was incorporated into the iron ore moisture measurement routine at TMPM’s physical-chemical analysis laboratory, which plays a crucial role in ore production and commercialization, and is a mandatory step according to ABNT (Brazilian Technical Standards Association), as presented in^20,21^ to ensure transportation safety. The laboratory’s operations are directly integrated into the industrial process, ensuring the experiments reflect real-world applications. The proposed equipment, designed as a bench-scale system, streamlines moisture analysis of sampled ore, enhancing efficiency by accelerating this critical step. Additionally, it provides valuable insights into the overall moisture trend of the transported ore, contributing to more effective process control and decision-making.

For the experiment, we used samples of IOCJ (Iron Ore Carajás) in the final product stage. At this phase, the ore has well-defined properties that vary in a well-defined range. These are established through regular physical-chemical analyses to meet client requirements regarding granulometry, iron content, and contaminant levels. These strict quality controls help standardize the moisture measurement process, minimizing errors.

According to data from the Brazilian National Waterway Transportation Agency (ANTAQ)^22^, IOCJ was responsible for 21.04% of all bulk cargo transported in 2023, totaling approximately 166.334 million tons. Given this high production volume, studying this specific material is relevant and justified, as it plays a significant role in the Brazilian economy.

We conducted the *in loco* experiment in two scenarios: compressing and not compressing the samples. We collected data on both scenarios, which were used to create two different ML models. We seek to achieve the objectives of our study, which include a) developing a methodology for measuring iron ore moisture with a lightweight ML algorithm, such as tree-based models, and b) investigating if ore compression affects moisture measurement. The ML model must be lightweight enough to run in an 8-bit microcontroller since we intend to embed it in the development of a commercial moisture analyzer.

The proposed approach, based on compressed ore samples and TinyML, achieved a mean absolute error of 0.12 percentage points (pp) and an expanded uncertainty of ±0.35 pp using a Decision Tree model. These results are sufficient to discriminate between safe and unsafe moisture levels, thereby providing practical value for industrial applications.

Therefore, we formulated two questions to guide our research. As a result, our methodology sought to answer the following research questions:

- **RQ1:** Which machine learning models achieve the best trade-off between accuracy, latency, and memory when measuring iron ore moisture in TinyML applications?
- **RQ2:** What is the impact of ore compression on the accuracy and reliability of machine learning-based moisture measurement systems?

The structure of this paper is organized as follows: Related Works provide an overview of existing studies and approaches that evaluate bulk material moisture. Fundamental Concepts present the capacitive principle of the proposed sensor. Methodology describes the instrumentation system’s design, functioning, and calibration. The results and analysis are discussed in Results, highlighting key findings of the experiment in the industrial environment. The research questions that guided this study are addressed in Discussion. Finally, Conclusion summarizes the contributions, discusses limitations, and proposes potential directions for future work.

## Related works

This section distinguishes off-line and on-line approaches to bulk-material moisture measurement. Off-line techniques include the traditional gravimetric method, loss-on-drying (LOD) moisture analyzers, and capacitive sensors. In contrast, online methods–such as infrared/hyperspectral systems and computer vision approaches installed on conveyor belts–provide continuous, in-process monitoring for control. Our contribution falls within the offline category, targeting rapid and repeatable measurements on compressed iron-ore samples for pre-shipment decisions.

Miljak et al.^23^ introduced an online moisture meter for iron ore that utilizes microwaves and operates on a conveyor belt. Similarly, Viana^12^ developed a system for measuring the moisture content of aluminum ore on a conveyor belt. Zhu et al.^24^ also applied microwaves to determine the moisture content of concentrated iron ore. Additionally, Indra et al.^25^ employed the microwave sweep method, combined with stratified random sampling, to evaluate the moisture content of nickel ore being transported on conveyor belts. These works address on-belt, online measurement under dynamic conditions (varying bed height and loading, particle-size segregation, temperature, and density). In contrast, our pre-shipping analyzer performs an offline measurement on compressed samples under controlled industrial conditions.

Li et al.^26^ integrated microwave spectrum analysis with machine-learning regression, evaluating several models. They also performed on-site validation on 27 groups of coal, reporting encouraging predictive performance. The authors conclude that the results were promising; however, further testing in real-world applications is needed to further validate their suitability for industrial use. Additionally, microwave instrumentation entails practical considerations, such as equipment cost and periodic recalibration, that can affect scalability in production settings^13^.

There are applications using computer vision. Tang and Esmaeili^27^ utilized an unmanned aerial vehicle (UAV) equipped with thermal imaging sensors to capture surface radiation data and then correlate it with surface moisture through an empirical linear regression model. However, the methodology is limited to outdoor, open areas and relies heavily on consistent solar radiation, requiring measurements to be taken in the afternoon for uniform surface temperatures. Weather conditions such as rain or cloudy days render the method unusable. An image-based approach was suggested by Zhou and Liu^14^. It evaluated the moisture of iron ore green pellets using image feature extraction and polynomial regression. However, it lacks industrial validation. Buchczik et al.^28^ developed a method for measuring the moisture content of fine copper ore, integrating computer vision and thermography techniques with a focus on evaluating the influence of particle size on water content determination. Computer vision techniques were employed to determine particle size, while the IR camera assessed moisture over a broad range of measurements and particle sizes. Three models – linear, quadratic, and spline regression – were evaluated on both uniform particle size samples and mixed granulometries. Although the results were promising, the IR method measures moisture only on the sample’s surface, and the experiment has not been validated in an industrial environment. Additionally, cameras face challenges in industrial settings due to dust, rain, and poor lighting conditions, which negatively impact image processing accuracy^1,15^.

The patent developed by Andrade et al.^29^ presents a method and system to determine the moisture content of iron ore transported on a conveyor belt. It uses hyperspectral cameras paired with computer vision and machine learning algorithms to identify the water content in the ore. However, hyperspectral cameras have a high acquisition cost and have challenges validating their use in industrial applications.

As offline approaches, there are the LOD analyzers for moisture determination^30^. However, they require heating cycles and a turnaround time of minutes, which limits sampling frequency immediately before shipping^31^. We propose a compressed capacitive method with embedded TinyML that provides a rapid offline pre-shipment check in the industrial laboratory, calibrated against LOD. Moreover, LOD analyzers determine moisture in less representative small subsamples (typically a few grams; instrument capacities are on the order of tens to at most  100–200 g), while the proposed instrument is capable of measuring samples of up to 700 g.

Lage et al.^16^ developed a bench system specifically for measuring the moisture of iron ore. The system operates on the principle of a current-to-voltage converter (I-V) with a trans-impedance amplifier to assess the relative permittivity of the ore, which is then used to determine its moisture content. For future developments, they propose using a manual press to compact the ore within the instrument chamber, thereby minimizing the effects of air gaps on capacitive sensor readings. However, as noted by Rego^18^, the I-V method may not be the most suitable for industrial applications where the material under test (MUT) has high conductivity due to its lack of precision.

An improvement in evaluating the permittivity of high-conductivity materials, such as ore, is the real-dual-frequency method (RDFM). This method effectively reduces the impact of high conductivity on relative permittivity measurement by using affordable low-frequency hardware^18^. Pinto et al.^17^ applied this approach with a capacitive sensor to measure moisture on a conveyor belt, while Santos et al.^32^ used it similarly to assess ore moisture in a train wagon prototype. However, neither application compared machine learning models nor experimented with measuring the ore’s moisture under compression.

Table 1 summarizes different works focused on ore moisture measurement. Our method uniquely utilizes a capacitive sensor combined with ore sample compression, leveraging a TinyML approach to estimate moisture content. Unlike previous studies, we conducted complex impedance measurements while compressing ore samples. Additionally, earlier research on capacitive sensors primarily focused on linear regression for moisture estimation. In contrast, we use multiple input features to evaluate lightweight tree-based machine-learning algorithms to enhance the instrument’s accuracy and ensure the regressor is suitable for execution on a microcontroller.

**Table 1:** Related Works Focusing on ore moisture measurement.

Work	Microwave	Computer vision	Capacitive sensor	Capacitive sensor + compression + TinyML
Miljak et al.^23^	$\bullet$	$\circ$	$\circ$	$\circ$
Viana^12^	$\bullet$	$\circ$	$\circ$	$\circ$
Zhu et al.^24^	$\bullet$	$\circ$	$\circ$	$\circ$
Indra et al.^25^	$\bullet$	$\circ$	$\circ$	$\circ$
Li et al.^26^	$\circ$	$\bullet$	$\circ$	$\circ$
Tang and Esmaeili^27^	$\circ$	$\bullet$	$\circ$	$\circ$
Zhou and Liu^14^	$\circ$	$\bullet$	$\circ$	$\circ$
Buchczik et al.^28^	$\circ$	$\bullet$	$\circ$	$\circ$
Andrade et al.^29^	$\circ$	$\bullet$	$\circ$	$\circ$
Lage et al.^16^	$\circ$	$\circ$	$\bullet$	$\circ$
Pinto et al.^17^	$\circ$	$\circ$	$\bullet$	$\circ$
Santos et al.^32^	$\circ$	$\circ$	$\bullet$	$\circ$
**Our work**	$\circ$	$\circ$	$\circ$	$\bullet$

## Fundamental concepts

This Section presents fundamental concepts of the capacitive principle of the proposed sensor. In electromagnetism, the complex permittivity describes the ability of a material to store and dissipate energy in response to an applied electric field. For a lossy dielectric medium, this property can be represented by:

1 $$\begin{aligned} \dot{\varepsilon } = \varepsilon _0 \varepsilon _r - j \frac{\sigma }{\omega }, \end{aligned}$$

where $\varepsilon _0$ is the permittivity of free space (=$8.854 \times 10^{-12} \; F \; m^{-1}$), $\varepsilon _r$ is the relative permittivity (dimensionless), $\sigma$ is the conductivity ($S \; m^{-1}$) and $\omega$ is the angular velocity ($rad \; s^{-1}$).

According to electrical circuit theory, considering the coupling of the capacitive sensor to the medium under test, the complex permittivity becomes related to the complex impedance as^17^:

2 $$\begin{aligned} \dot{Z} = k_g^{-1} [\sigma ^{-1} - j (\omega \varepsilon _0 \varepsilon _r)^{-1}], \end{aligned}$$

where $k_g$ is the geometric constant of the capacitor (m). Hence, the impedance can be rewritten as:

3 $$\begin{aligned} \dot{Z} = G^{-1} - j (\omega C)^{-1}, \end{aligned}$$

where *G* is the conductance (S), which is the inverse of resistance ($G = R^{-1}$) and *C* is the capacitance (F).

The complex impedance can be measured using the two plateaus’ current-to-voltage circuit using a transimpedance amplifier, which excites the sensor with sinusoidal signals at two distinct frequencies and calculates the impedance by measuring each voltage gain^17,33^. The system’s frequency response exhibits a characteristic curve with two well-defined plateaus and a 20 dB/dec interval between them. The first plateau, corresponding to low frequencies, allows the measurement of the material’s conductance by suppressing the capacitive effect. The second plateau, at high frequencies, enables the determination of the capacitance while minimizing the influence of conductance.

In 3, as angular frequency $\omega$ increases, the influence of conductance on the total impedance diminishes. In this regime, the capacitive effect becomes dominant, making it more straightforward to measure. However, the plateau-based approach exhibits limitations when applied to lossy media, as the requisite frequency range expands proportionally with the material’s conductivity. Consequently, the associated circuitry becomes increasingly costly and complex at higher frequencies. To overcome this challenge, the measurement methodology RDFM incorporates a mathematical model of the capacitive sensor’s frequency response. This approach enables accurate permittivity measurements at comparatively lower frequencies than traditional methods, even for material under test with high conductivity^18,19^.

## Methodology

### Equipment operational principles

The measurement system incorporates a capacitive moisture sensor and a method for standardizing ore compression, consisting of these key components:

- **Measuring Chamber (1):** Made from acrylic, this box-shaped chamber with a hollow center and no top contains the stainless steel electrodes (2) and holds the ore sample for measurement. Additionally, a ruler is included for measuring the ore height, allowing volume determination.
- **Electrodes (2):** Two stainless steel plates are attached parallel to the interior sides, forming the capacitive sensor with floating terminals, meaning neither electrode is grounded. The electrodes are 48 mm apart and have a geometric constant of 0.083 m.
- **Metallic Support Structure (3):** Provides the framework for the compression system, ensuring stability during operation.
- **Press (4):** Serves as a surface for compressing the ore sample, aiding in consistent measurements.
- **Threaded Spindle and Digital Torque Wrench (5):** Enables vertical displacement of the compression system and includes a torque wrench to standardize the applied force.
- **Load Cell (6):** Measures the sample mass, allowing for bulk density determination.
- **Hood-Shaped Niche (7):** Designed to house a support block, this feature prevents overloading of the load cell during the ore compression process.
- **Measurement Circuit Box (8):** Houses the electronics and circuitry necessary for the measurement process. The complex impedance measurement employs an 8-bit microcontroller to implement the RDF method, as described by^17,18^.

**Fig. 1:**
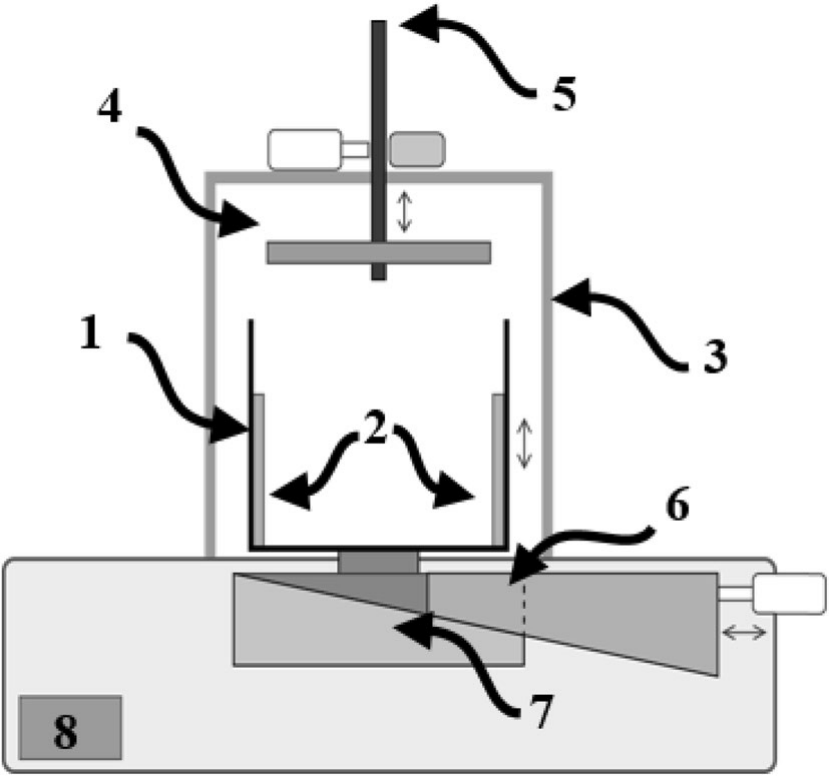
Schematic drawing of the bench equipment for ore moisture measurement.

**Fig. 2:**
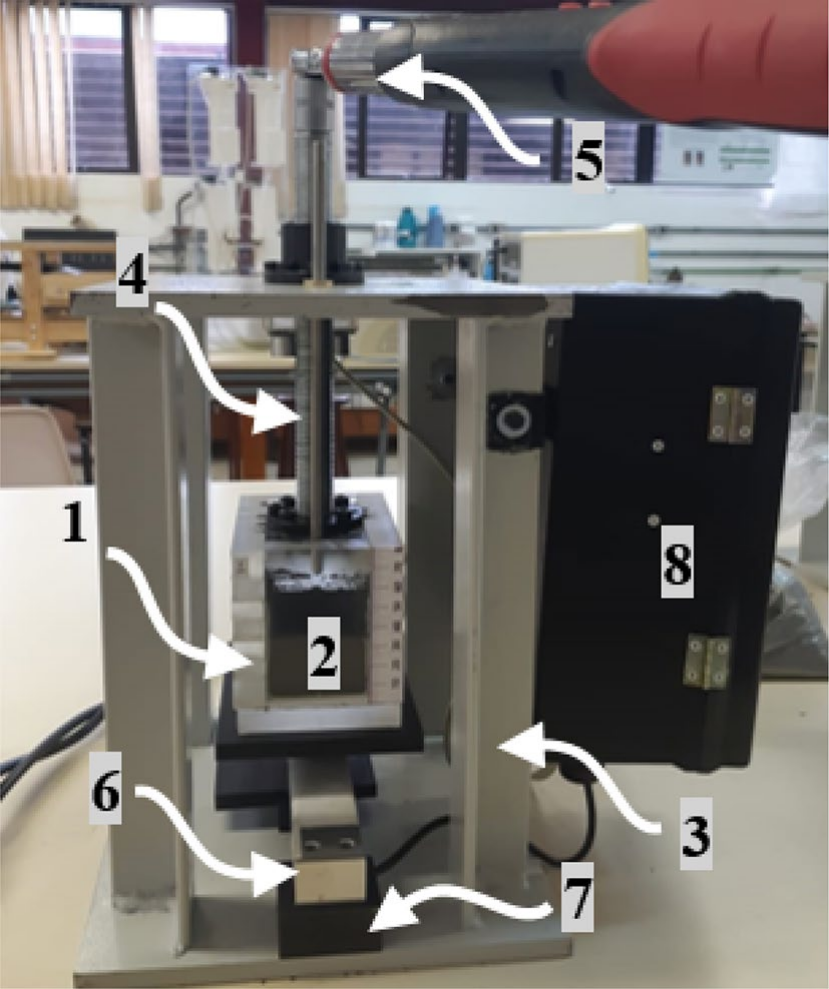
Real prototype of the bench equipment for ore moisture measurement.

Figure 1 shows the schematic drawing of the setup, while Figure 2 presents the prototype used during the measurement campaign. The equipment operating methodology involves placing an approximately 700 g IOCJ sample in the measuring chamber, weighing it, and measuring its initial complex impedance before starting the compression process. After each compression, the complex impedance is re-measured in a continuous cycle until the operator reaches five compressions, and then the process can be restarted.The equipment’s grounded metallic structure shields the sensor from external electromagnetic fields, while the climate-controlled environment minimizes concerns about temperature and humidity variations.

The accuracy of the sensor in measuring relative permittivity and conductivity was verified using aqueous substances as references (Table 2). Conductivity measurements at 20 °C were obtained using a Del Lab DL-150P conductivity meter. These substances, including mixtures of water and alcohol and varying concentrations of water and NaCl, were used to cover a range of relative permittivity and conductivity values. It is important to note that our measurement system achieved the highest accuracy for conductivity values $>90\,\upmu \hbox {S/cm}$, which encompasses the range observed in iron ore samples. The higher relative errors observed for very low conductivity samples ($<10\,\upmu \hbox {S/cm}$) are outside the primary application range of this instrument. The complex impedance measurement circuit used in the equipment is the same as presented by^17,18^, deploying the RDFM on an 8-bit microcontroller with 500 kHz and 8 MHz signals applied to the sensor.

**Table 2:** Reference Samples.

Samples	Reference $\varepsilon _{r}$	Measured $\varepsilon _{r}$	Reference $\sigma (\mu S/cm)$	Measured $\sigma (\mu S/cm)$
Air	1.0	1.8	0.07	7.63
Ethyl alcohol	25.2	22.3	4.33	5.40
25% water + 75% ethyl alcohol	39.3	37.0	4.28	1.61
50% water + 50% ethyl alcohol	54.0	54.8	4.03	3.19
75% water + 25% ethyl alcohol	69.6	69.6	4.83	8.55
Distilled water	80.2	77.6	3.34	10.76
Drinking water	80.2	78.2	26.11	13.45
Water + NaCl (1)	80.5	77.4	90.90	91.41
Water + NaCl (2)	80.3	82.5	266.50	260.70
Water + NaCl (3)	80.0	79.7	432.00	431.74
Water + NaCl (4)	80.0	80.1	851.30	853.15

### Iron ore moisture measurement

The second part of the sensor calibration involves collecting data from different but moisture-known iron ore samples. We carried out *in loco* tests for 6 days at the Ponta da Madeira port of Vale SA located in São Luiz, Maranhão. We used real ore samples called Iron Ore Carajás (IOCJ), which represent about 90% of all material loaded at the port. It was used 55 ore samples with different moisture percentages. All these samples were analyzed before ship transportation.

The partial IOCJ samples used in the experiment come from five different shipments. These samples exhibit a *Fe* variation between 64.87 and 65.89%, $SiO_{2}$ between 1.38 and 1.66%, $Al_{2}O_{3}$ between 0.87 and 1.44%, *Mn* between 0.07 and 0.16%, *P* between 0.05 and 0.07%, *CaO* between 0.01 and 0.03%, *MgO* between 0.03 and 0.05%, S between 0.008 and 0.012%, PPC between 2.97 and 3.99%, $TiO_{2}$ between 0.078 and 0.114%.

It is important to note that while all samples used in this study are classified as the same product type (IOCJ sinter feed), the observed variations in measurement results cannot be primarily attributed to data noise. Our sensor validation with homogeneous substances demonstrates high measurement repeatability with minimal standard deviations^17,18^. Rather, the variability in our iron ore moisture measurements is derived from three main factors: (1) natural compositional differences between samples collected from different loading lines, with variations in iron content, silica, alumina, and other constituents, as detailed above; (2) the initial arrangement of particles within the measuring chamber before compression, which affects the initial complex impedance readings; and (3) the variable effectiveness of compression in eliminating air gaps due to differences in particle size distribution.

**Fig. 3:**
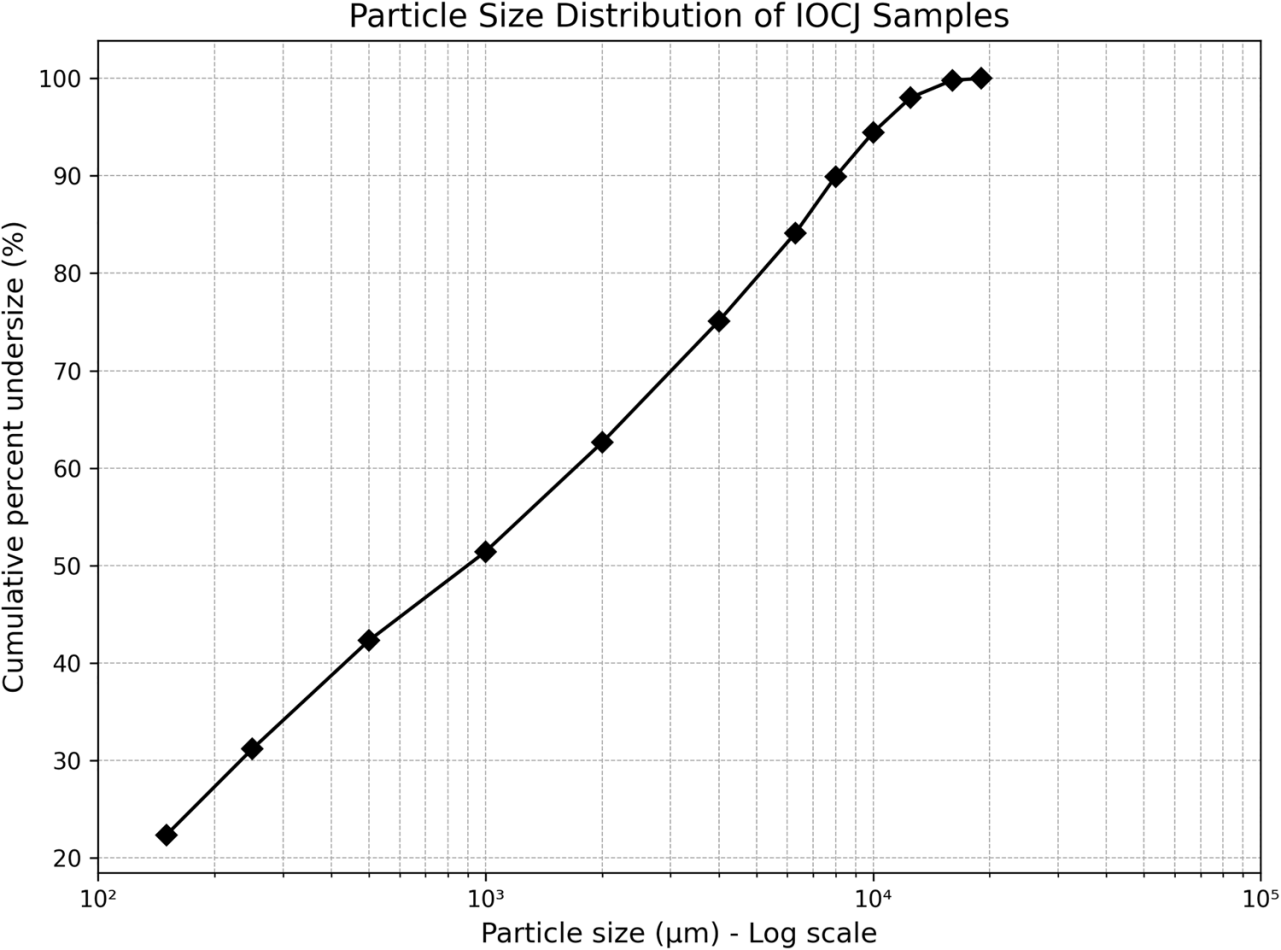
Particle size distribution of IOCJ samples showing the cumulative percent undersize as a function of particle size ($\mu$m).

Vale’s physical analysis procedure includes the determination of particle size down to an aperture size of 150 µm, with results showing that, on average, 77.65% of the particles are coarser than this size, while 22.35% pass through the 150 µm sieve. The compression process, although standardized by torque measurements, interacts differently with samples exhibiting distinct granulometric profiles. This behavior supports our observation that sample compression enhances measurement accuracy by reducing, though not entirely eliminating, material-dependent variations. Figure 3 presents the average particle size distribution (PSD) curve of the samples, plotted as cumulative percent undersize versus particle size (µm). The results indicate a progressive increase in cumulative undersize with decreasing particle size, showing that most of the material is concentrated in the finer fractions. This distribution is consistent with the expected granulometric behavior of iron ore, where a significant portion of particles is below 2 mm. Although the D_50_ values ranged from 0.5 mm to 1.0 mm, small pebbles up to 16 mm in diameter were occasionally observed, contributing to notable granulometric heterogeneity within individual samples.

The Vale SA analysis laboratory uses gravimetric techniques^8^ standardized to evaluate the ore moisture. We used these values as the ground truth of our models. Furthermore, we collected data without compressing the sample and then gradually compressing it. In this process, we measured variables such as electrical conductivity, relative permittivity, density, torque, and chamber level.

**Fig. 4:**
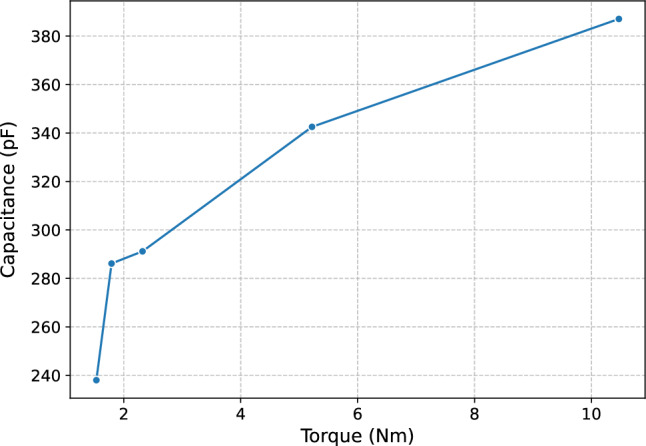
Capacitance as a function of torque..

The measurement process with iron ore begins when we place the sample of the mineral in the measuring chamber, a load cell determines its mass, and we put the chock into the hood-shaped niche. Initially, we measure the variables without compression. Then, we apply torques of approximately 1.5 Nm, 1.7 Nm, 2 Nm, 5 Nm, and 10 Nm to the ore sample. The choice of torque was made empirically to ensure measurement accuracy while preserving the integrity of the acrylic chamber, which has a maximum limit of 15 Nm. Since greater compression reduces empty spaces in the sample, improving the precision of complex impedance measurement, we set a safe limit of 10 Nm to prevent structural damage and variations in electrode spacing. Figure 4 illustrates the variation of sensor capacitance with applied torque, highlighting how increasing compression influences the dielectric response.

The moisture associated with the evaluated ore samples was between 7.0% and 9.5%, considering the wet base. These values represent common moisture for the rainy season and represent a large part of the company’s measurement of interest range. Table 3 shows the summary of the statistics of the dataset.

**Table 3:** Descriptive statistics of the dataset.

Variable	Unit	Mean	Std. Dev	Min	Max
Torque	Nm	3.99	3.20	1.52	10.80
Level	mm	64.83	3.92	55.40	76.10
Density	g/cm³	2715.17	138.52	2129.95	3077.37
Conductivity	µS/cm	55.70	15.83	25.87	109.66
Relative Permittivity	-	49.94	16.32	19.34	93.97
Moisture	%	8.36	0.46	7.03	9.49

We developed two datasets–one with the measurements without compression and another with compression, consequently with variations on the applied torque. For model development, it is crucial to have a lightweight algorithm, as it must operate on microcontrollers, which characterizes a TinyML application.

Given the deployment target on microcontrollers (TinyML constraints), we prioritized methods with modest computational and memory footprints. We evaluated five supervised regressors:

(i) Multiple Linear Regression (MLR) ^34^,
(ii) a Decision Tree (DT) ^35^, and
(iii) Random Forests (RF) ^36^ with 5, 10, and 15 trees, denoted RF5, RF10, and RF15, respectively.

These models differ in how they use variables: MLR assumes linear additivity of predictors, whereas DT/RF capture interactions and nonlinearities through hierarchical splits and ensembling, respectively.

To assess generalization and guard against overfitting, we employed Leave-One-Out Cross-Validation (LOOCV). In LOOCV, each sample serves once as the test case while the remaining $n-1$ samples are used for training; the held-out prediction $\hat{y}_i^{(-i)}$ is compared with the reference $y_i$. This protocol provides an essentially unbiased, sample-level estimate of out-of-sample performance; overfitted models would exhibit low training error but elevated LOOCV error. Although computationally intensive, LOOCV is advantageous for small datasets because it maximizes the training data per split^37,38^. All reported metrics are computed only from the LOOCV predictions: Mean Absolute Error (MAE), Mean Squared Error (MSE), coefficient of determination $R^2$, and an uncertainty estimated as twice the standard deviation of the residuals .

We consider the input variables $X=\{\text {Torque (Nm)},\,\text {Level (mm)},\,\text {Density (g\,cm}^{-3}),\,\text {Conductivity } (\upmu \text {S/cm)},\,\text {Relative} \text {Permittivity}\}$ and the output variable $y=\text {Moisture (\%)}$. To ensure fairness and interpretability, all models were trained on the same input set $X$ and evaluated under the identical LOOCV protocol. This design avoids confounding due to model-specific pre-selection of variables and provides a transparent comparison of predictive performance. The code and data are available in the GitHub repository^39^.

We selected the algorithm with lower error and best performance on a microcontroller, addressing the **RQ1**. We used the 8-bit microcontroller ATmega2560, running at 16 MIPS, to embed the models. This choice was made due to its common availability, cost-effectiveness, and suitability for transforming models into C code for microcontroller deployment. This was done by means of Python *micromlgen* library, which converts machine-learning models from Python into C implementations. Each model was deployed to the microcontroller, and key metrics such as processing time, flash memory usage, and RAM consumption were recorded. We chose the algorithm that best performs on the prediction.

To address **RQ2**, we trained two models: one with sample compression and the other without. We compared their performances to investigate the impact of ore compression on moisture measurement. Performance metrics were calculated for each model, and statistical tests were performed for comparison.

## Results

### Model development

We employed leave-one-out cross-validation for training and testing on the dataset with ore’s compression and calculated metrics such as MSE, MAE, R², and uncertainty. Table 4 shows the obtained metrics for each model.

**Table 4:** Performance metrics of machine learning models. MAE and uncertainty are reported on the original moisture scale (percentage points) using LOOCV predictions..

Model	MSE	MAE	R²	Uncertainty
MLR	0.0826	0.2058	0.5568	±0.58
DT	0.0302	0.1241	0.8211	±0.35
RF5	0.0288	0.1308	0.8266	±0.34
RF10	0.0318	0.1283	0.8221	±0.36
RF15	0.0283	0.1301	0.8464	±0.34

**Fig. 5:**
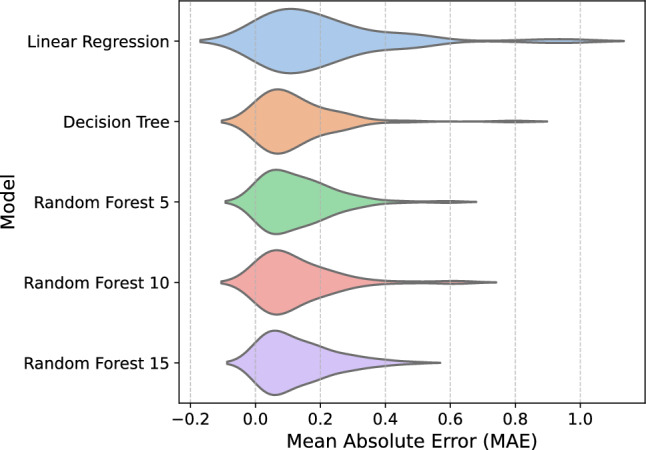
Violin plot of the Mean Absolute Error (MAE) for each model.

The violin plot of the Mean Absolute Error (MAE) for each model is presented in Fig. 5. Except for multiple linear regression, the models obtained very similar behavior. We also observed that the RF15 exhibited less variability compared to the others. Hence, we performed a Welch’s t-test to determine whether the RF15 is statistically different from the others. Table 5 shows that Random Forest 15 significantly outperforms Linear Regression; however, according to the test, there are non-significance differences between other algorithms. Thus, the algorithms with non-significance differences were evaluated by their computational costs.

**Table 5:** Paired t-test conclusions for model comparisons.

Comparison	Conclusion
RF15 vs MLR	Reject null hypothesis
RF15 vs DT	Fail to reject null hypothesis
RF15 vs RF 5	Fail to reject null hypothesis
RF15 vs RF 10	Fail to reject null hypothesis

### Evaluation of microcontroller performance

We have collected metrics related to the microcontroller process during its functioning. The processing time refers to the duration taken by the microcontroller to execute a single prediction. Flash memory usage indicates the amount of non-volatile storage required to house the model code and associated data. RAM consumption measures the dynamic memory used during the model’s execution.

**Fig. 6:**
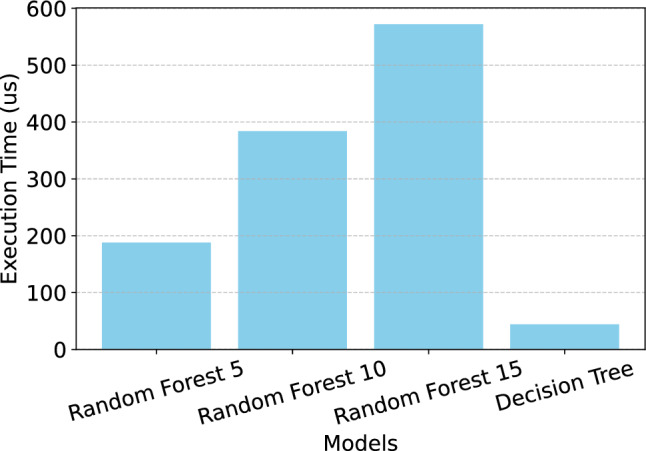
Execution time comparison.

The decision tree algorithm exhibits a slight increase in flash and RAM usage when compared to the random forest algorithms. However, it demonstrates a significantly lower execution time. Fig. 6 presents the execution time comparison for each model evaluated. Since the t-test revealed no significant differences in error rates between the decision tree and the other algorithms, this suggests that the decision tree is more efficient for this task .

### Impact evaluation of ore compression

We selected the decision tree algorithm to examine the effects of sample compression on moisture measurement. Using data gathered from non-compressed ore samples, we developed another decision tree model. To evaluate the non-compressed model, we also used LOOCV. During each evaluation phase, we calculated metrics to assess the performance of the processes. The outcomes of these evaluations are detailed in Table 6.

**Table 6:** Comparison of models based on compressed versus non-compressed ore samples. MAE and uncertainty are reported on the original moisture scale (percentage points) using LOOCV predictions..

Metric	Compressed	Non-compressed
MAE (pp)	0.12	0.47
Uncertainty (pp)	$\pm 0.35$	$\pm 1.3$
$R^{2}$	0.82	0.54

**Fig. 7:**
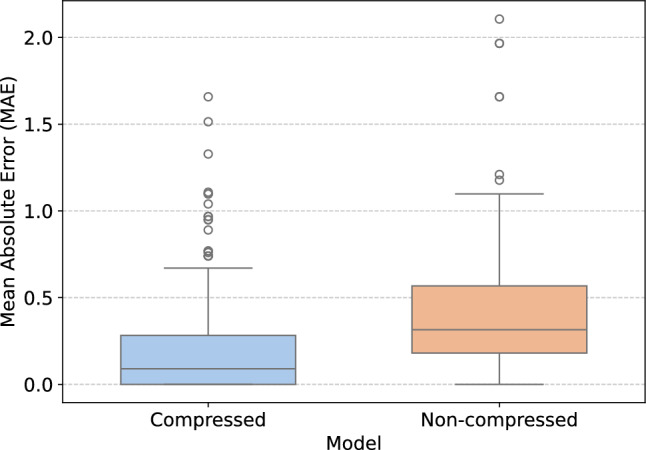
Box plots representing the evaluation results of the compressed and non-compressed models, showing the distribution of prediction errors (MAE) for each case.

The compressed model demonstrates superior performance, achieving a substantially lower MAE of 0.12 pp compared to 0.47 pp for the non-compressed model. This indicates that compressing ore samples leads to more accurate predictions on average. Moreover, the uncertainty of the compressed model is markedly lower, suggesting that its predictions are more consistent and reliable. The higher $R^2$ value further supports its effectiveness, showing that the compressed model explains a larger proportion of the variance in the data. Overall, these metrics highlight the advantage of applying compression to ore samples. Figure 7 presents the box plot of MAE for the two models, emphasizing the performance differences between them.

The Mann-Whitney U Test was conducted to compare these groups and assess the significance of their differences. The results indicate a statistically significant difference between the two groups, as evidenced by a U-statistic of 1137.5 and a p-value of 6.061e-13. This suggests a meaningful difference in the median values of the two groups under study. The result allows us to reject the null hypothesis that there is no difference between the groups, supporting the alternative hypothesis of a significant difference in their central tendencies. These highlight the significant improvement in the measurement of compressed ore samples.

**Fig. 8:**
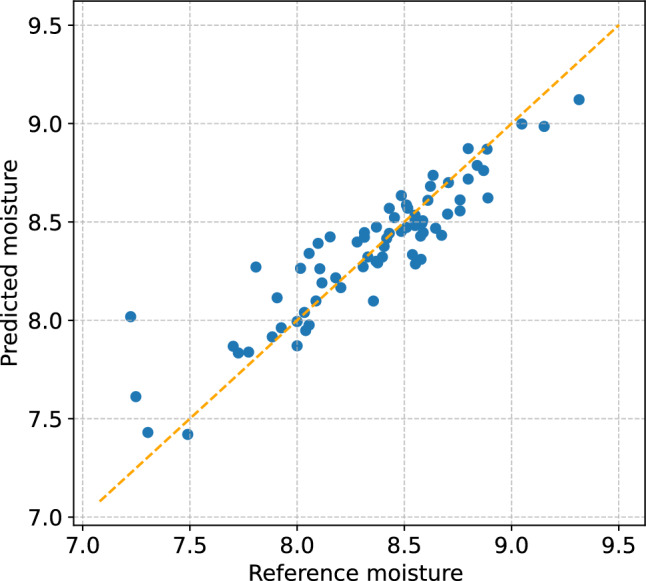
Plot of the model for compressed samples, showing predicted and reference moisture values across all data points..

Figure 8 presents the model for compressed samples: leave-one-out predictions versus reference moisture values. Each point corresponds to a sample, plotted with the reference moisture content on the x-axis and the model prediction on the y-axis. The orange dashed line represents the line of equality $y = x$ , where perfect agreement would occur. The majority of points are distributed closely around the equality line, indicating that the model predictions are largely unbiased across the studied range. This confirms that compression improves model stability and that the predictive performance is sufficient to discriminate safe versus critical moisture levels.

## Discussion

A response can be given to the research questions based on the results obtained.

**RQ1:** Which machine learning models achieve the best trade-off between accuracy and latency when measuring iron ore moisture in TinyML applications?

Previous work has explored the use of multiple linear regression; however, it did not yield satisfactory results in our experiments. As a result, we trained and validated other machine-learning algorithms. We tested decision trees and random forests using configurations of 5, 10, and 15 trees. These tree-based algorithms demonstrated significant improvement compared to multiple linear regression. When comparing the performances of the different algorithms, we found that their results were quite similar. However, the random forests with 15 trees achieved slightly better outcomes. To verify if there are statistical differences between the ML error populations, we performed a t-test, which failed to reject the null hypothesis. This indicates that, based on prediction errors, the algorithms are statistically similar. Hence, we embedded each model on the ATmega2560 and collected the microcontroller’s performance metrics, such as execution time, free RAM, and flash memory consumption. RAM and flash showed no significant changes, but the execution time of the decision tree was much lower than the others. Consequently, we chose the decision tree algorithm due to its efficiency based on the prediction’s errors and response latency.

**RQ2:** What is the impact of ore compression on the accuracy and reliability of machine learning-based moisture measurement systems?

We compare two decision tree models: one trained on compressed ore samples and another on non-compressed samples. The compressed dataset demonstrates significant improvements over the non-compressed dataset across all evaluated metrics. Using compressed samples leads to a 73.76% reduction in MAE, indicating substantially higher accuracy. The uncertainty is reduced by 73.33%, reflecting enhanced reliability in the predictions. Furthermore, the R² shows a 52.00% improvement, underscoring the superior ability of the compressed dataset to explain variance in the data. These results collectively highlight the advancements in precision, consistency, and predictive capability of compressing samples when measuring complex impedance for moisture prediction.

## Conclusion

The work presents a bench system for measuring iron ore moisture using complex impedance with standardized compressions. It aims to improve decision-making in the iron ore production chain, particularly during ship loading, by providing a real-time, high-accuracy measurement method. Currently, the mineral industry lacks such a method, which affects decision-making. The study proposes a solution by compressing ore samples and utilizing the RDFM method along with a TinyML model to assess moisture content.

The proposed system, tested with IOCJ samples at Vale SA’s Ponta da Madeira port, demonstrated that compression significantly improves prediction accuracy, achieving an average MAE of 0.12 pp and reducing error by 73.76% compared to non-compressed samples. In addition, decision-tree models enabled near-instant predictions (44 µs), cutting the turn-around time from hours, as in conventional methods, to microseconds.

These results indicate that the method not only ensures high precision but also allows real-time decision-making during ship loading, reducing delays and associated costs. Faster and more accurate measurements can increase loading capacity, improve scheduling, and enhance global competitiveness at high-volume ports.

This work marks significant progress in transforming the technology into industrial equipment. Given TMPM’s high cargo volume, faster measurements can enhance loading capacity, reduce costs, and improve global competitiveness. Although the equipment is calibrated for a single ore product, establishing reliable technology at one of Latin America’s largest ports presents a key opportunity to maximize efficiency. As moisture measurement speed and accuracy are crucial in the loading process, an improved system can greatly accelerate this step.

For future work, we aim to further enhance the hardware of the bench system to make it fully automated, improving efficiency and reducing the need for manual intervention. Concerning the compression process, we intend to use industrial servomotors designed for precise control of torque, speed, and position, ensuring a robust and reliable solution to maintain process consistency and enhance the equipment’s trustworthiness.

We also plan to assess model update techniques, as different types of ore necessitate specific model calibrations, and to address model degradation. We will evaluate incremental learning methods, such as just-in-time learning, since traditional moisture measurements provide ground truth data, even though they respond slowly.

## Data Availability

All data generated or analyzed during this study are included in this published article.
